# Dental Treatments under General Anesthesia on Children with Special Health Care Needs Enrolled in the Spanish Dental Care Program

**DOI:** 10.3390/jcm10020182

**Published:** 2021-01-06

**Authors:** María Pilar Pecci-Lloret, Julia Guerrero-Gironés, Belén López-González, Francisco Javier Rodríguez-Lozano, Daniel Oñate-Cabrerizo, Ricardo E. Oñate-Sánchez, Miguel R. Pecci-Lloret

**Affiliations:** Special Care in Dentistry and Gerodontology Unit, Meseguer Hospital, Faculty of Medicine, University of Murcia, 30100 Murcia, Spain; mariapilar.pecci@um.es (M.P.P.-L.); Belen.lopezg@um.es (B.L.-G.); fcojavier@um.es (F.J.R.-L.); forraw@gmail.com (D.O.-C.); reosan@um.es (R.E.O.-S.); miguelramon.pecci@um.es (M.R.P.-L.)

**Keywords:** general anesthesia, dental treatments, children with special health care needs, dental public programs, postoperative complications

## Abstract

The purpose is to analyze the medical characteristics of children with special health care needs (CSHCN) recommended for dental treatment under general anesthesia (GA), postoperative complications, and dental treatment outcomes under the regulation of the Spanish Dental Care Program (PADI). 111 clinical records were selected. The study population was divided into three age groups. The quantitative data was specified as the mean ± SD. For the qualitative variables, the Chi-Square test was used. One-way ANOVA and Bonferroni tests were used to examine the effect of the “age group” and the number of treatment procedures. A total of 1473 treatment procedures were performed, of which 110 (7.5%) were cleanings, 898 (61%) were restorative procedures, 332 (21.7%) were extractions, 22 (1.6%) were endodontic treatments, 62 (4.2%) were pulpotomies, and 59 (4%) were stainless steel crowns. Regarding the mean number of incisor root canal treatments (RCT), age group 3 received a significantly higher mean number of incisor RCTs than age group 1 (*p* = 0.02). Age group 1 received a higher average of pulpotomies and stainless-steel crowns (*p* = 0.00) compared to groups 2 and 3. GA is a safe procedure for the dental treatment of CSHCN, with minimal postoperative complications, which should be included among dental public programs.

## 1. Introduction

Children with special health care needs are considered by the American Academy of Pediatric Dentistry (AAPD) as those who have “any physical, developmental, mental, sensory, behavioral, cognitive, or emotional impairment or limiting condition that requires medical management, health care intervention, and/or use of specialized services or programs” [1]. The estimated prevalence of children with special health care needs (CSHCN) ranges from 13–18% of the population under 18 years of age in developed countries [2]. In the U.S., 13% of children and adolescents aged 17 and younger have a special need for healthcare [3].

In the last decade, several strategies to decrease the risk of caries have been implemented with preventive success. However, CSHCN still show limitations in maintaining a good oral health, early childhood caries being highly prevalent [4]. Furthermore, special diets, medications, and oral motor habits like atypical swallowing, lip sucking, or tongue interposition, can favor the development of dental complications for many CSHCN [3]. There are various methods for the evaluation of caries risk among this group of patients. For example, there are caries risk assessment tools provided by the AAPD and American Dental Association (ADA), and other protocols such as the Caries Management by Risk Assessment (CAMBRA). Nonetheless, these tools differ in the parameters and criteria used for the caries risk assessment, and, consequently, the categorization of patients that is provided also differs. Remarkably, CSHCN under 14 years of age are categorized as high risk (ADA) and as moderate over 14 years of age, while CAMBRA only considers children under five years of age with “developmental problems” as being high risk [5]. Although CSHCN have similar or superior preventive dental programs, others factor such as ethnicity, age, or socioeconomic status could influence the use of preventive dental services and programs [6].

CSHCN with extensive dental complications tend to exhibit anxiety and a lack of cooperation, mainly due to physical limitations, mental disabilities, or behavioral management problems. Altogether, these factors make conventional dental treatment and oral examinations extremely challenging for the dental practitioners [7]. In these patients, under general anesthesia (GA), we are able to overcome the patients’ cognitive, motor, and sensory factors and could thus complete the diagnosis and perform dental treatments with less difficulty and a higher quality [8]. Additionally, recent evidence indicates that parental satisfaction with dental treatment under GA has been continuously increasing over the recent years and is now accepted more favorably than other active or passive behavioral management techniques [9].

Although GA is a safe procedure, postoperative dental morbidity or complications have been described, dental pain and bleeding being the most common complications [10]. In addition, GA is an expensive procedure performed by trained anesthetists in hospital facilities, and it requires a time-consuming preoperative intervention for both the dental practitioners and primary caregivers of CSHCN. Furthermore, this group of patients may also require reinterventions or further treatment under GA [11].

However, there are a few previous studies in Spain about the different dental treatment modalities under GA in CSHCN. Thus, the aim of this study was to analyze the medical characteristics of CSHCN recommended for treatment under GA, postoperative complications, percentage of GA reinterventions, and dental treatment outcomes under the regulation of the Spanish Dental Care Program for Children (PADI).

## 2. Methods

One hundred and eleven clinical records of CSHCN enrolled in the PADI Program of the Region of Murcia treated between the period of January 2008 and December 2019 by two specialist doctors and their respective teams at the specialized centers “Clínica Belén” and “Hospital Quirónsalud” (Murcia, Spain) were selected. This oral health program for CSHCN includes children from six to 14 years old. The sample size analysis was calculated using the website www.openepi.com with a confidence interval of 95% and power of 80%.

PADI is a dental care system for children and adolescents. It is based on four fundamental concepts: (1) public financing, (2) mixed provision by public centers and private centers, (3) preventive protocols before dental treatment, and (4) payment to the private sector for capitation to general care and, through an agreed fee, to special care like dental trauma and malformations or like CSHCN treatments under GA. Currently, eight of the 17 Spanish autonomous communities, including the Region of Murcia, apply to the PADI program, where it is widely accepted. The PADI system covers 39% of the Spanish population between six and 15 years of age. The system is not homogeneous, and significant differences are apparent between the communities.

This retrospective epidemiological study was approved by the Research Ethics Commission of the University of Murcia (ID: 2827/2020), obtaining the necessary information by collecting data presented in the medical records. All of the parents/legal guardians of the involved patients signed a written informed consent before carrying out the dental treatments under a general anesthesia on their children/authorized representatives. They were also informed about the possible use of their data for future scientific research.

Patients were referred to our service and were included in the PADI program of the Region de Murcia. They are from any of the municipalities in this Spanish province. The CAMBRA method was used for the caries risk assessment. All patients treated under general anesthesia were considered at high risk due to having active cavities, evident bacterial plaque, and taking various medications that decreased salivary flow. After every general anesthesia, patients were in the hospital for at least 12 h. The children were reevaluated a week after the intervention, again a month later, and subsequently had periodic check-ups with their usual dentists every three or every six months.

The study population was divided into three groups, based on age: 6–8 years (*n* = 48), 9–11 years (*n* = 39), and 12–14 years (*n* = 40). The following variables were recorded: Date of birth, gender, medical conditions or diseases, dental or medical postoperative problems, date on which the general anesthesia was requested and performed, dental treatments carried out, preoperative panoramic radiography, and whether or not reports were missing from the patients’ medical history, so as to know that the medical data was not provided to the dentists but instead was presented to the hospital anesthetist in the preoperative examination.

The data from the medical records used were entered into Cliniwin Software (Qüentin Informática SL, Valencia, Spain), an online medical record management program for medical professionals. Information was imported into an Excel database (Version 16.0 Microsoft Office, Redmond, WA, USA) for further statistical analysis. The processing and analysis of the results were carried out with the SPSS statistical software version 20.0 (SPSS, Inc., Chicago, IL, USA). Quantitative data was specified as the mean ± standard deviation (SD). For the qualitative variables, the Chi-Square test was used, with the Yates correction, if necessary. A one-way ANOVA was used to examine the effect of the “age group” and the number of treatment procedures. Post hoc multiple comparisons were performed using the Bonferroni method. The level of statistical significance adopted was *p* < 0.05.

## 3. Results

A total of 111 CSHCN (74 males and 37 females) that had received dental treatment under GA between 2008 and 2019 were included in the final analysis. The mean age of the CSHCN at the time they received treatment under GA was 9.69 ± 2.738 years, with a range from six to 14 years. The mean number of days from when GA was requested until it was performed was 81.78 ± 53.35 days. The range from 50 to 75 days for the waiting time was the most frequent.

Panoramic radiographs were obtained from 31 of them (27.9%), while the remaining 80 patients (72.1%) were treated without this preliminary radiographic examination. Besides this, 27 patients did not have complete medical reports (24.3%). Regarding the number of times that patients received treatment under GA, 86 patients (77.5%) received dental treatment under general anesthesia once, 23 patients (20.7%) twice, and two patients (1.8%) three times. Therefore, 127 interventions under GA were performed on a total of 111 patients studied.

**Figure 1:**
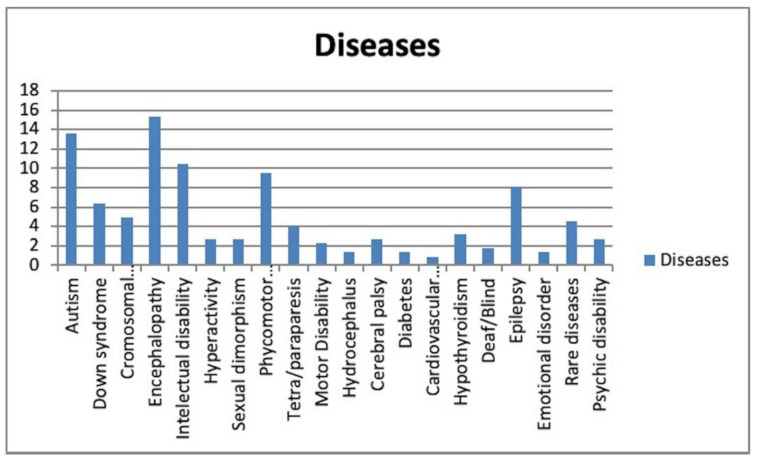
Distribution of the CSHCN based on their medical conditions.

The distribution of the CSHCN based on their medical conditions is shown in Figure 1. Subjects with encephalopathy received general anesthesia most frequently in terms of the percentage relative to the total number of subjects (15.4%).

**Figure 2:**
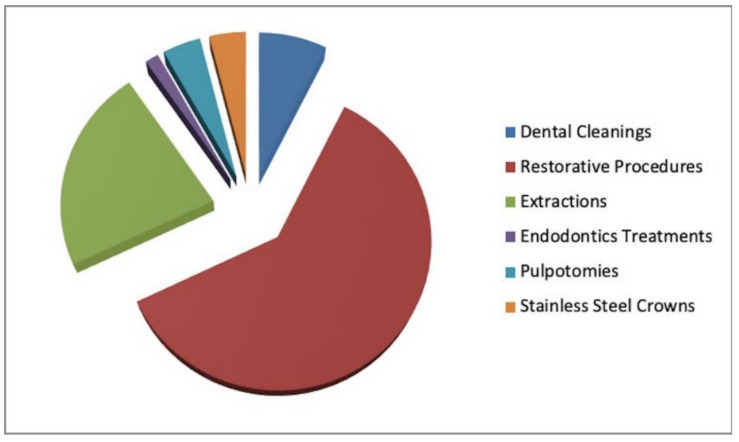
Treatment procedures performed under general anesthesia from 2008 to 2019.

A total of 1473 treatment procedures were performed on the CSHCN under GA over the 11-year period, of which 110 (7.5%) were dental cleanings, 898 (61%) were restorative procedures (resin composite fillings), 332 (21.7%) were simple extractions, 14 (1%) were root canal treatments in incisors, one (0.1%) was a root canal treatment in a premolar, seven (0.5%) were root canal treatments in molars, 62 (4.2%) were pulpotomies, and 59 (4%) were stainless steel crowns. Therefore, most of the procedures performed under GA were restorative in nature, with composite fillings being the most frequently performed treatments (Figure 2). The mean number of restorative treatment procedures per child was 8.09. A statistically significant correlation was observed between fillings and extractions (*p* = 0.001), which meant that when one increased the other decreased and vice versa; and between pulpotomies and crowns (*p* = 0.000).

A total of 230 treatment procedures were performed on the CSHCN under a repeated GA, which was a second or third GA administration on the same patients. Among them, 22 (9.6%) were dental cleanings, 143 (62.1%) were restorative procedures (composite resin), 54 (23.5%) were simple extractions, eight (3.5%) were root canal treatments in incisors (incisor RCT), and three (1.3%) were root canal treatments in molars (molar RCT). There were no significant differences between the mean number of treatments between the first GA interventions and the repeated GA (*p* = 0.124).

The CSHCN were divided into three age groups: 6–8 years (Age Group 1), 9–11 years (Age Group 2), and 12–14 years (Age Group 3). Based on the different age groups, the distributions of the CSHCN were 34.8% (6–8 years), 23.7% (9–11 years), and 17.8% (12–14 years), respectively. No statistically significant difference was observed between genders across the different age groups (*p* = 0.71). The mean number of treatment procedures for each age group is shown in Table 1. Regarding the mean number of incisor RCTs, age group 3 received a significantly higher mean number of incisor RCTs than age group 1 (*p* = 0.02). Age group 1 received a higher average number of pulpotomies (*p* = 0.00) compared to groups 2 and 3. Finally, the use of stainless-steel crowns was significantly higher in age group 1 (primary dentition) than in age group 2 (mixed dentition) or age group 3 (permanent dentition) (*p* < 0.05).

With regards to the most prevalent medical diseases in these patients over the years, given the low number of subjects per disorder, only those with the highest frequencies were taken into account, since they were the only valuable data for the research: for example, autism (*n* = 32) and Down’s syndrome (*n* = 16). After Pearson’s Chi-square test, it was found that there were no significant differences between the proportion of children with autism and Down’s syndrome in relation to the interventions under GA received over the years, with a *p*-value = 0.082. Mean tooth extraction in the autistic population was significantly lower than for the nonautistic population (*p* = 0.035). Patients with Down’s syndrome received a significantly higher mean number of dental cleanings than the rest of the patients (*p* = 0.001).

The intubation for GA was performed either nasally or orally. Nasal intubation was used for 98.43% of the patients. In 100% of performed GA interventions there were no postoperative complications, either medical or dental. All patients were discharged within 12–15 h after the general anesthesia (Table 2).

## 4. Discussion

To our knowledge, this is the first study that reported information about dental treatments under nonconventional anesthesia within the regulation of the Spanish Dental Care Program for Children (PADI).

In our study, there was a disproportion regarding the gender of the patients. (74 men and 37 women). Some previous studies with CSHCN supported this data distribution, although it is not clear why the masculine gender often outnumbers the feminine gender [12,13,14].

The most frequent age for performing GA in CHSCN was between six and 7.5 years of age. This could be explained by the fact that the PADI Program benefits begin at six years of age and by the increasing awareness of parents regarding the importance of maintaining good oral hygiene in their children. It may also be due to the promotion of oral health by dentists [15].

The impossibility of performing panoramic radiographs in our work had a high prevalence among the study sample (72.1%). Schabl et al. [16] explains this difficulty by the physical and/or mental disability of certain patients, which prevents them from keeping their heads in a stable and firm position during the time of exposure required for the radiographic examination. In these cases, clinicians may be forced to reach a presumptive diagnosis and make treatment decisions during the interventions [17]. Lo Giudice et al. affirmed that the diagnostic phase could be improved by measuring IL-6 in saliva because the levels were significantly higher in children with active caries than in children without caries [18]. New instruments such as intraoral scanners and CAD-CAM technologies have improved the clinical phase of treatment, such as through the intraoperative performance of a crown or inlay [19].

Our findings show that 20.7% of patients had to receive a second intervention under GA, and they even had to receive a third one in 1.8% of cases. In a similar study performed by Mitchell et al., it was reported that, from a total of 96 studied patients, the percentage of patients who received repeated treatments under GA was 7.2% [20]. Similarly, Roeters et al. [21] reported repeated GA interventions in 10.2% of cases from a total of 248 patients. Berkowitz et al. [22] found that 3% of 84 patients with special needs who were under study were treated a third time under general anesthesia. Therefore, it is essential to establish a follow-up protocol to avoid having to go through a second or third general anesthesia procedure. At the same time, parents and caregivers of CHSCN should receive appropriate health care education and commit to prevent this from occurring [23].

Regarding the period from when general anesthesia was requested until it was carried out, we found a waiting time ranging from 50 to 75 days. None of the GA interventions were performed urgently; all of them were scheduled. The economical, organizational, and infrastructural requirements for the procedures under GA may explain the considerable duration of the waiting time. Furthermore, the number of clinicians qualified to provide care to this group of patients is still limited in the Region of Murcia. Previous studies reported the importance of prioritizing elective dental treatments under GA through a system based on the medical and dental risks of CSHCN. This system would allow urgent cases to be carried out as soon as possible and would reduce the waiting list for treatments under GA [24,25].

Encephalopathy was the most prevalent disease (15.4%). A clear difference was seen regarding chromosomal disorders (5%), hydrocephalus (1.4%), and diabetes (1.4%). Similar results were found in a study by Akpinar et al. [26], in which the researchers categorized different diseases suffered by patients into five groups (Group I: Down syndrome; Group II: Other syndromes; Group III: Psychiatric disorders such as autism; Group IV: Physical disabilities such as blindness or deafness, and Group V: Mental or motor disorders that include cerebral palsy, hydrocephalus, encephalopathies...) in a sample of 1045 cases, obtaining a higher prevalence of cases belonging to Group V (*n* = 629), followed by Group III (*n* = 121) and Group I (*n* = 120).

The treatment rates for the restorative procedures performed under GA for CSHCN were higher than all of the other procedures (61%), and the mean number of restorative treatment procedures performed per child was 8.09. This treatment approach was similar to previous studies. For example, Mallineni et al. [9] reported that restorations represented 47% out of a total of 3217 procedures performed on 275 patient samples with special needs over 10 years. In addition, Peretz et al. [13] showed that from 121 patients, the treatment rates for restorative procedures were 7.6 per patient.

Regarding the treatments performed under repeated GA, similar findings were obtained to the treatments carried out in the first interventions: most of the performed treatments were fillings, followed by extractions and dental cleanings. Guidry et al. [11] described how children treated in the first intervention with composites and with a lower number of sealants and extractions were more likely to receive a second intervention under GA in the following four years. Similarly, Oh et al. [7] reported that patients who received irregular follow-up visits were four times more likely to undergo a second intervention than those who received periodic visits. Furthermore, Kakaounaki et al. [27,28] showed that from a total of 484 children who received GA, 10.7% subsequently received at least one unplanned repeated GA, with dental caries being a factor for the reintervention in 84% of the cases.

In our study, no postoperative complications were observed, either medically or dentally, and patients were discharged 12–15 h after the intervention. In a similar manner, Caputo et al. [29] demonstrated that the incidence of mortality in patients with special needs was minimal and that the impact of morbidity was limited to minor events. Therefore, from the available data, it can be stated that GA is a safe and successful procedure for the performance of dental treatments in CSHCN.

Among the limitations of the present study, the lack of medical data from various patients could have influenced the statistical analysis, as the missing information could have altered the values of the studied variables. In some cases, significant results could not be obtained because the sample size was insufficient. Last, as the patients considered in this study are enrolled in the PADI program, follow-up visits are not carried out by the team performing the treatments under GA but by the patients’ usual dentist, and thus the data about the follow-up evaluations could not be obtained.

Finally, from the findings observed in the present study, we want to highlight the need for public programs that include dental prevention measures and treatments under general anesthesia that are accessible to all patients with a recognized disability, with no age limit. On the other hand, more clinicians with a specialized training in special care in dentistry are needed to assist this group of patients.

Based on the findings from the retrospective evaluation of clinical records, GA is a safe and useful procedure for the dental treatment of children with special health care needs, with minimal postoperative complications, which should be included and standardized among dental public programs.

## Figures and Tables

**Table 1:** Values are means ± standard deviation. Groups identified by different superscripts were significantly different (*p* < 0.05).

	Age Group		
Treatment procedures	6–8 Years	9–11 Years	12–14 Years
Dental cleanings	0.87 ± 0.34 ^a^	0.91 ± 0.29 ^a^	0.96 ± 0.20 ^a^
Restorative procedures	7.11 ± 2.96 ^a^	6.91 ± 3.28 ^a^	7.79 ± 3.98 ^a^
Incisor RCT	0.00 ± 0.00 ^a^	0.03 ± 0.18 ^a^	0.21 ± 0.59 ^b^
Molar RCT	0.00 ± 0.00 ^a^	0.06 ± 0.24 ^a^	0.13 ± 0.34 ^a^
Extractions	3.15 ± 3.92 ^a^	2.00 ± 2.83 ^a^	2.29 ± 2.29 ^a^
Pulpotomies	1.19 ± 1.64 ^a^	0.19 ± 0.47 ^b^	0.00 ± 0.00 ^b^
Stainless steel crowns	1.15 ± 1.61 ^a^	0.16 ± 0.45 ^b^	0.00 ± 0.00 ^b^

RCT: root canal treatment; same superscripts between groups (^a^ and ^a^) indicate *p* > 0.05; different superscripts between groups (^a^ and ^b^) indicate *p* < 0.05.

**Table 2:** Summary of the studied parameters.

Studied Parameters		Quantity	Percentage
Panoramic Radiograph	YES	31	27.9%
	NO	80	72.1%
Medical reports	Incomplete	27	24.3%
	Complete	84	75.7%
General Anesthesia received	Once	86	77.5%
	Twice	23	20.75%
	Three times	2	1.8%
Postoperative Complications	YES	0	0%
	NO	111	100%
Treatments	Dental cleanings	110	7.5%
	Restorative procedures	898	61%
	Extractions	332	21.7%
	Endodontics treatments	22	1.6%

## Data Availability

The data presented in this study are available on request from the corresponding author. The data are not publicly available due to privacy reasons.
